# Bridging the Microplastics–Public Health Research Gap: A Call for Translational Action in Vulnerable Populations

**DOI:** 10.1002/hsr2.71050

**Published:** 2025-07-21

**Authors:** Md. Mahadi Hassan, Noushin Nohor

**Affiliations:** ^1^ Department of Public Health and Informatics Jahangirnagar University Savar Bangladesh; ^2^ aiHealth Lab, Center for Health Innovation, Research, Action, and Learning–Bangladesh Dhanmondi Bangladesh; ^3^ Population Health Studies Division Center for Health Innovation, Research, Action, and Learning–Bangladesh Dhanmondi Bangladesh

## Conflicts of Interest

The authors declare no conflicts of interest.

## Transparency Statement

The lead author, Md. Mahadi Hassan affirms that this manuscript is an honest, accurate, and transparent account of the study being reported; that no important aspects of the study have been omitted; and that any discrepancies from the study as planned (and, if relevant, registered) have been explained.

To the Editor,

We have read with great interest the article by Alvitez et al. (2025) titled “Current Trends, Spatio‐Temporal Dynamics of Microplastics Research and Global Public Health: A Scientometric Study” [1]. The authors provided an impressive scientometric analysis of global research trends on microplastics and their implications for public health. This study offers a valuable overview of the thematic evolution, key contributors, and geographic collaborations shaping this emerging field. While their work effectively highlights the academic momentum behind microplastic research, it also inadvertently exposes a pressing concern: the lack of context‐specific studies that translate into meaningful health interventions, particularly in low‐ and middle‐income countries (LMICs).

One of the most striking findings from the study is the dominance of high‐income countries in publication output and scholarly impact, despite LMICs often bearing a disproportionate burden of environmental exposure and health vulnerability. For example, Bangladesh, a densely populated country with rampant plastic pollution and fragile waste management systems, has been involved in just a handful of collaborative publications in this field. This disparity underscores an urgent need for increased funding and capacity‐building to foster localized, high‐quality research in affected regions.

Furthermore, while the article briefly touches upon indoor exposure and the intersection with COVID‐19, it does not delve deeply into the health outcomes or pathophysiological pathways through which microplastics may affect vulnerable populations, such as children, pregnant women, or individuals with pre‐existing respiratory conditions. Microplastics in air, water, and food have been associated with inflammation, oxidative stress, and even neurodegenerative outcomes. Yet, there remains a glaring absence of epidemiological studies linking microplastic exposure to clinical endpoints, particularly in real‐world settings where co‐exposures to other environmental toxins are common [2, 3, 4]. The current scientometric trends, as revealed by the authors, show increasing interdisciplinary collaboration and publication volume. However, quantity does not equate to impact if it fails to inform public policy and health interventions. There is a critical need to translate research outputs into regulatory standards for plastic use, urban planning, and public health advisories, especially in megacities across South Asia and Sub‐Saharan Africa, where exposure risks are intensifying.

Finally, we commend Alvitez et al. for documenting the thematic shift of microplastics research toward public health. This is an encouraging sign that the scientific community is beginning to move beyond ecotoxicology and into the domain of population health. The next imperative, however, is to prioritize longitudinal, community‐based studies that assess cumulative exposure, bioaccumulation, and chronic health impacts. Without such efforts, the scientific knowledge amassed over the last 5 years risks remaining in the academic sphere—unapplied, unregulated, and ultimately, unhelpful to those most at risk.

## Author Contributions

**Md. Mahadi Hassan:** conceptualization, writing – original draft, writing – review and editing. **Noushin Nohor:** writing – review and editing. All authors have read and approved the final version of the manuscript.

## Ethics Statement

The authors have nothing to report.

## Consent

The authors have nothing to report.

## Data Availability

The authors have nothing to report.
